# Pre-existing mental health disorders and fear of COVID-19 pandemic: Data from a phone survey in community-dwelling older adults recruited in the NutBrain study

**DOI:** 10.3389/fpsyt.2022.995308

**Published:** 2022-11-07

**Authors:** Elena Perdixi, Sara Bernini, Silvia Conti, Nithiya Jesuthasan, Matteo Cotta Ramusino, Alfredo Costa, Federica Prinelli

**Affiliations:** ^1^Unit of Behavioral Neurology and Dementia Research Center, IRCCS Mondino Foundation, Pavia, Italy; ^2^Epidemiology Unit, Institute of Biomedical Technologies, National Research Council, Segrate, MI, Italy

**Keywords:** fear, COVID-19, mental health disorders, neurocognitive disorders, depressive symptoms, phone survey, older people

## Abstract

**Background:**

COVID-19 has caused a parallel epidemic of fear, anxiety, depression, stress, and frustration, particularly among the most fragile and vulnerable individuals, such as older people and those with previous mental health disorders. The present study aims to investigate the association between pre-existing mental health disorders, particularly depressive symptoms and Mild Cognitive Impairment (MCI), and the fear of COVID-19 and to explore which cognitive domains were involved in coping with fear in older people.

**Materials and methods:**

In April 2020, we conducted a phone-interview questionnaire on community-dwelling older adults living in Lombardy Region (Italy) who participated in the NutBrain study. At baseline, socio-demographic characteristics along with lifestyles, and medical history were recorded. Participants underwent a neuropsychological battery exploring the global cognitive function and specific cognitive domains, to detect cases of MCI. The Center for Epidemiologic Studies Depression scale (CES-D) was used for screening depressive symptoms. During the phone survey, respondents were assessed using a structured questionnaire querying about fear of the COVID-19 pandemic. We performed multivariate logistic regression models to study the association between MCI and depressive symptomatology and fear. We also explored which cognitive domains were associated with fear. Odds Ratios (OR) with Confidence Intervals (95%CI) were estimated adjusting for potential confounders.

**Results:**

Out of the 351 respondents (mean age 73.5 ± 6.1 years, 59.8% women, 49.1% high education), at baseline, 22.9% had MCI and 18.8% had depressive symptoms. In the multivariate analyses gender, age, and body mass index were significantly associated with the fear score. Considering different domains of fear, MCI was associated with fear of being infected themselves (OR 2.55, 95%CI 1.39–4.70) while depressive symptoms were associated with fear of contagion for family members (OR 2.38, 95%CI 1.25–4.52). Impaired executive cognitive function was positively associated with the highest tertile of the fear score (OR 3.28, 95%CI 1.37–7.74) and with fear of contagion for themselves (OR 3.39, 95%CI 1.61-7.17).

**Conclusion:**

Older adults experienced different fear reactions, particularly when suffering from neurocognitive disorders and depressive symptoms; executive dysfunction was associated with increased fear. These results highlighted the need to pay attention to the psychological effects of the outbreak of COVID-19 to target intervention, especially among vulnerable subgroups of individuals.

**Clinical trial registration:**

[ClinicalTrials.gov], identifier [NCT04461951].

## Introduction

The outbreak of the novel coronavirus disease (COVID-19) can be considered one of the worst pandemics in the recent century (1). Since the beginning of the epidemic, older adults and people with serious comorbidities appeared particularly vulnerable to developing severe complications that could lead to hospitalization and/or death (2). In addition to the health problems, COVID-19 has caused a parallel epidemic of fear, anxiety, depression, stress, and frustration in people of all ages (3). In particular, symptoms related to the anxious-depressive sphere have occurred among the most fragile and vulnerable individuals, such as older people and those with previous mental health disorders (4). These persons had to face difficulties such as isolation, illness, distance from loved ones and difficulty in understanding what was going on: this created psychological reactions to the pandemic, one of which is fear (5). Fear is a biological and psychological construct that derives from a series of environmental and behavioral stimuli; it is a state of the organism in reaction to a dangerous stimulus (6). During the COVID-19 pandemic, dangerous stimuli were: unknown diseases and discouraging updates about contagions and deaths. Fear of COVID-19 involves, among others, fear of being infected by the virus, as well as fear for household and family members (7). Previous studies reported that older people and those suffering from psychiatric comorbidities and mental disorders showed more fear of COVID-19 (8) and were likely to be more afraid of COVID-19 due to higher vulnerability to stress compared with the general population (9).

COVID-19 pandemic-related measures, including changes in routine activities, restrictions, and isolation have negatively impacted psychological, cognitive, and neuropsychiatric spheres in people with dementia or cognitive impairment (10–13). Indeed, for people with neurocognitive disorders it is important to have routines, an active social life, leave the house and be engaged in productive activities that can stimulate different cognitive skills. The inability of the reduction about these actions created a sense of disorientation: many of these people were isolated, without interaction with family members and experienced a sense of helplessness and growing fear (14).

Furthermore, levels of COVID-19-related fear were found positively associated with other psychological factors such as depressive and anxiety symptoms and risk perception of COVID-19 among older people, suggesting the significant effect that COVID-19 has on psychological well-being and mental health (5).

Although fear of COVID-19 may aggravate pre-existing conditions such as neurocognitive disorders and depressive symptoms and can increase psychological distress and anxiety symptoms among older people (15, 16), little attention has been paid to these aspects among this susceptible group of individuals. As far as we know, no previous studies investigated the role of pre-existing mental health disturbances, in terms of depressive symptoms and neurocognitive deficits, and which cognitive domains were involved in coping with the fear of COVID-19 in older people. To fill this gap, we used data collected through a phone interview among older participants of the observational NutBrain (*Nutrition, gUT microbiota, and BRain AgINg*) Study (17) to examine the association of depressive symptoms and cognitive performance with the fear of COVID-19.

## Materials and methods

### Study design, setting and population

NutBrain^1^ is an ongoing population-based study of non-institutionalized community-dwelling older individuals aged 63-94 years residing in Lombardy Region (Italy). The details of recruitment and study procedures were described elsewhere (17). Briefly, at baseline (started in 2019) socio-demographic characteristics along with lifestyles, functional status, and medical and drug history were collected by using validated scales and *ad hoc* questionnaires. In addition, participants underwent an extensive neuropsychological assessment to investigate global cognitive functioning and different cognitive domains for identifying suspected cases of Mild Cognitive Impairment (MCI). Those individuals with a cognitive profile suggestive of MCI underwent a subsequent clinical examination including a neurological visit to confirm the diagnosis. Due to the pandemic, the study recruitment was interrupted at the beginning of March 2020, thus from April 1 to 22, 2020 the participants who accepted to be re-contacted after baseline assessment were interviewed by phone by trained personnel to collect information about their health status. 387 older adults were recruited at baseline and were contacted by phone. Of them, 10 did not answer or were not contactable and 26 refused to participate because not interested, resulting in a final sample of 351 individuals analyzable (response rate 91%).

### Data collection at baseline

The variables collected during the extensive baseline assessment were also included in the present analysis as follows. S*ocio-demographic variables:* age (continuous), gender, education (categorized as high school or higher, middle school, elementary school or less), occupation (blue collar vs. white collar), and living arrangement (living alone vs. not living alone). *Lifestyle variables:* the frequency of engagement in leisure activities was collected through the Cognitive Reserve Index questionnaire (CRIq) (18). Leisure activities were grouped into three categories as: mental (reading books or newspapers, driving a car, using the smartphone or pc and engaging in artistic activities); social (being part of associations, going to the cinema or theater, traveling, taking care of grandchildren or pets); and physical (house working, sporting, gardening) (19). The frequency of engagement in each activity was grouped in tertiles, a leisure activity score was created by summing up mental, social and physical activities and then categorized in low, moderate and high. Smoking habits were classified as never and former or current smokers. *Clinical variables:* functional evaluations of activities of daily living were assessed using the Katz Index of Independence in Activities of Daily Living (ADL) (20) and the Instrumental Activities of Daily Living scale (IADL) (21), polypharmacy (more than 5 drugs per day, as a proxy of comorbidities), and body mass index (calculated as weight/height^2^ ratio, continuous). Depressive symptoms were assessed using the 20-item Center for Epidemiologic Studies Depression scale (CES-D) (22). Response options range from 0 to 3 for each item referring to the previous week: 0 = rarely or none (less than one day), 1 = some or little of the time (1–2 days), 2 = moderately or much of the time (3–4 days), 3 = most or almost all the time (5–7 days). The scoring of positive items (n. 4, 8, 12, 16) was reversed. The possible range of scores is 0 to 60, with higher scores indicating greater depressive symptoms. The standard cut-off point of 16 or more was used to classify individuals with depressive symptomatology (23). According to Albert’s criteria (24), MCI was defined as the presence of subjective cognitive complaints and objective cognitive impairment in one or two neuropsychological tests; impairment has to be greater than expected for an individual’s age and education levels, without impairment in activities of daily life (ADL and IADL). The neuropsychological profile was assessed using a well-established neuropsychological battery exploring the global cognitive function and specific cognitive domains (24) (Supplementary material). All the raw scores in the neuropsychological tests were corrected for age, gender, and education and compared with the values available for the Italian population (25). The corrected scores were firstly classified into equivalent scores on a 5-point ordinal scale (26) ranging from 0, meaning impaired, to 4, meaning normal. The equivalent scores were then reversed and dichotomized into normal (from 1 to 4 = 0) and impaired (0 = 1). The neurologist assigned a final clinical diagnosis of MCI after reaching an agreement between the neuropsychological and clinical examinations.

### Data collection during the COVID-19 phone interview

Trained interviewers – the same who conducted the baseline in-person assessment – performed the telephone interview extracted from the web-based EPICOVID19 38-item questionnaire developed by a team of experts during the first wave of the pandemic in Italy (March 2020) (27). The phone interview is reported in the Supplementary Annex 1. In particular, participants were asked if, since March 2020, they had experienced any of the following COVID-19–related symptoms: fever (> 37.5 degrees Celsius for at least three consecutive days); headache, chest pain, myalgia, olfactory and taste disorders, shortness of breath, and tachycardia; gastrointestinal disturbances (diarrhea, nausea, and vomiting); conjunctivitis; sore throat, rhinorrhea, and cough (all dichotomized as present/absent). Information regarding nasopharyngeal swab test results (categorized as not performed, performed with a negative result, performed with a positive result, and performed with an unknown result), hospitalization for confirmed or suspected infection (dichotomized as yes/no) contacts with suspected or confirmed COVID-19 cases (yes vs. no), flu and anti-pneumococcal vaccination (yes vs. no), use of drugs during the last two months (anti-inflammatory, anxiety/sleeping pills, supplements, antibiotics, anti-allergies), were also collected. Fear of COVID-19 was operationalized with three questions with 5 possible answers as 0 = no, 1 = just a little, 2 = neutral, 3 = quite enough, 4 = yes, a lot, asking participants how worried they were about contagion (i) compared with peers, (ii) for themselves, and (iii) for their household or family members. A total fear score was computed by summing the values from the three questions and ranged from 0 to 12, where the higher the scores, the greater the fear of COVID-19. The score was then categorized in tertiles as 1 = ≤ 4, 2 = 5 −7, and 3 = 8+ to explore set of patterns in the continuous variable and making easier the comparison of groups of individuals with low, medium or high levels of fear.

A dichotomous classification of the three questions related to fear was also created by collapsing numbers 0, 1, and 2 into 0 = none or low level of fear and numbers 3 and 4 into 1 = medium-high level of fear (28). We considered as refusals the interviews in which participants refused to participate in the first phone call, or when three phone calls attempted on different days and times were unanswered.

### Statistical analysis

Participants’ characteristics by tertiles of the fear score were described using mean (standard deviation – SD) for continuous variables and frequency (%) for categorical ones. ANOVA for continuous variables and the Chi-square tests for categorical variables were used to compare differences in participants’ characteristics. We estimated the odds ratios (ORs) and 95% Confidence Intervals (CIs) by using the multinomial logistic regression model to study the association of depressive symptoms and MCI with fear tertiles. Binary logistic regression models were also performed to evaluate the associations between depressive symptoms and MCI and the three different domains of fear. The potential confounders of the two set of models were selected based on theoretical knowledge and empirical criteria (*P* < 0.05 in univariate analysis) and included gender, age, educational level, occupation, smoking status, polypharmacy, body mass index, anti-pneumococcal vaccine, and leisure activities engagement. We further explored which cognitive tasks were involved in coping with fear. Firstly, we analyzed the distribution of impaired neuropsychological test scores across tertiles of fear and different fear domains. Secondly, when statistically significant differences in univariate analysis were observed, logistic regression models were carried out by including neuropsychological test scores (dichotomized variables, impaired vs. normal) in the model. All the analyses were performed using Stata 15.0 version (StataCorp LP, College Station, Texas, USA) and IBM SPSS Statistics for Windows version 25.0 (IBM Corp., Armonk, NY). A two-sided *P* < 0.05 was considered statistically significant. The tests were not corrected for the multiple comparisons given the exploratory nature of the study.

## Results

**FIGURE 1:**
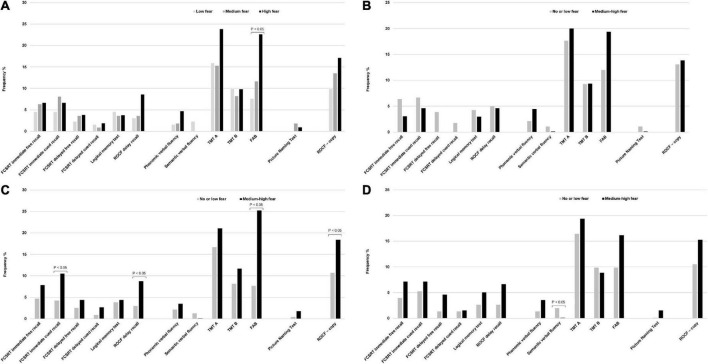
**(A)** Frequency of impaired neuropsychological tests and tertiles of fear score. **(B)** Frequency of impaired neuropsychological tests and fear of contagion compared with peers. **(C)** Frequency of impaired neuropsychological tests and fear of contagion for themselves. **(D)** Frequency of impaired neuropsychological tests and fear of contagion for family members. P-value denotes a statistically significant difference between normal and impaired test at the 0.05 level, as derived from chi-squared tests. Data are percentages. FCSRT immediate free recall, Free and Cued Selective Reminding Test immediate free recall; FCSRT immediate cued recall, Free and Cued Selective Reminding Test immediate cued recall; ROCF delayed recall, Rey-Osterrieth Complex Figure Test delayed recall; TMT A, Trail Making Test A; TMT B, Trail Making Test B; FAB, Frontal Assessment Battery; ROCF – copy, Rey-Osterrieth Complex Figure Test – copy.

Table 1 summarizes the baseline characteristics of the study sample consisting of 351 respondents according to the tertiles of the fear score (mean score 5.7 ± 3.1 SD). The mean age was 73.5 years ± 6.1 SD, 59.8% were women, 49.1% had a high educational level, 54.3% were blue-collar, 75.5% were not living alone, 22.9% had a diagnosis of MCI and 18.5% had depressive symptoms (mean CES-D 9.6 ± 0.4 SD). Compared with individuals in the lowest tertile of fear, those in the highest tertile were more likely to be women (*P* = 0.002), never smokers (*P* = 0.033), had a higher BMI, were less engaged in leisure activities during the lifespan and reported more depressive symptoms (borderline statistical significance). Table 2 reports the variables collected during the phone call according to the tertiles of the fear score. Rhinorrhea (12.3%), sore throat (10.8%), cough and myalgia (10.5%) were the fourth most common self-reported COVID-19-like symptoms. Supplements (38.2%) and anti-inflammatory drugs (35.9%) were the two most used classes of drugs since the beginning of the pandemic. 62.7% and 9.4% of the sample were vaccinated for flu and anti-pneumococcal, respectively. Only five individuals were hospitalized for COVID-19, 0.9% were tested for COVID-19 and only 1 participant was positive, 6.3% reported having contacts with suspected or confirmed positive cases of COVID-19. Participants in the highest tertile of fear mostly reported rhinorrhea (*P* = 0.005), headache (*P* = 0.009), tachycardia (*P* = 0.032), and used more anti-inflammatory drugs (borderline statistical significance). Multinomial logistic regression (Table 3) demonstrated that age (aOR 0.93, 95%CI 0.88–0.98) was inversely associated with high levels of fear. On the contrary, women (aOR 1.04–3.51) and increased body mass index (aOR 1.06, 95%CI 1.00–1.14) were positively associated. We also found a borderline statistically significant inverse association with high engagement in leisure activities (aOR 0.51, 95%CI 0.25–1.03) and former or current smoking habit (aOR 0.58, 95%CI 0.32–1.04). No statistically significant association with neurocognitive outcomes and depressive symptoms was observed. Table 4 reports the results of the binary logistic regression models. More than half of the study sample reported fear of contagion for family members, one-third reported fear for themselves and almost 20% fear of contagion compared with peers. The anti-pneumococcal vaccine was positively associated with fear of contagion compared with peers (model A, aOR 2.60, 95%CI 1.14–5.90). Age (aOR 0.94, 95%CI 0.89–0.98) and high engagement in leisure activities (aOR 0.46, 95%CI 0.23–0.85) reduced the probability of having fear of contagion for themselves while higher BMI (aOR 1.08, 95%CI 1.02–1.14) and having a MCI diagnosis (aOR 2.55, 95%CI 1.39–4.70) were associated with a high level of fear (model B). Fear of contagion for family members (model C) was inversely associated with age (aOR 0.94, 95%CI 0.90–0.98) and positively associated with the anti-pneumococcal vaccine (aOR 3.09, 95%CI 1.23–7.74) and depressive symptoms (aOR 2.38, 95%CI 1.25–4.52). A borderline statistically significant association was observed with former or current smoking habit (aOR 0.64, 95%CI 0.39–1.03). Figures 1A–D reported the frequency of impaired neuropsychological test scores vs. normal values relative to the total fear score and the three different domains of fear. P-value denotes a statistically significant difference between normal and impaired test at the 0.05 level, as derived from chi-squared tests. Performance at Frontal Assessment Battery (FAB), investigating executive functions, was significantly impaired among individuals in the highest tertile of fear score (**1A**). The Free and Cued Selective Reminding Test (FCSRT) immediate cued recall, which is a sub-score of Free and Cued Selective Reminding Test (verbal episodic long term memory), Rey-Osterrieth Complex Figure Test (ROCF) delay recall (visuospatial episodic long-term memory) and copy (visuospatial abilities), and FAB were significantly impaired among individuals with high fear of contagion for themselves (**1C**). Those with high fear for family members had impaired semantic verbal fluency (**1D**). Supplementary Tables 1, 2 show the results of the multinomial and binary logistic regression models that include those neuropsychological tests that resulted statistically significantly different in univariate analysis. FAB was positively associated with total fear score (aOR 3.28, 95%CI 1.37–7.74, high vs. low tertile) (**S1**) and with fear of contagion for themselves (aOR 3.39, 95%CI 1.61–7.17) (**S2**). No other statistically significant associations were found.

**TABLE 1:** Baseline characteristics of participants by tertiles of fear score (N = 351).

Characteristics at baseline	Low (score ≤ 4) *N* = 102, 22.6%		Medium (score 5–7) *N* = 106, 30.2%		High (score 8+) *N* = 103, 22.8%		*P-value*	All *N* = 351	
Women	64	48,5	71	62,8	75	70,8	*0,002*	210	59,8
Age, years (mean, SD)	74,0	6,5	73,4	5,9	73,1	5,9	*0,567*	73,5	6,1
Education							*0,164*		
High school or more	72	54,5	56	50,0	44	41,5		172	49,1
Middle school	40	30,3	37	33,0	34	32,1		111	31,7
Illiterate or primary school	20	15,2	19	17,0	28	26,4		67	19,1
Occupational status							*0,581*		
White collar	69	47,7	53	47,3	44	41,5		160	45,7
Blue collar	69	52,3	59	52,7	62	58,5		190	54,3
Living arrangement								235	67,0
Not living alone	105	79,5	84	74,3	76	71,7	*0,354*	265	75,5
Living alone	27	20,5	29	25,7	30	28,3		86	24,5
Smoking status							*0,033*		
Never	64	48,5	60	53,1	69	65,1		193	55,0
Former or current	68	51,5	53	46,9	37	34,9		158	45,0
Body mass index (mean, SD)	26,8	4,3	26,7	4,3	27,9	5,0	*0,068*	27,1	4,5
Polypharmacy (>5 drugs/day)	24	18,2	27	23,9	30	28,3	*0,178*	81	23,1
Leisure activities							*0,069*		
Low	39	29,5	45	39,8	49	46,2		133	37,9
Moderate	43	32,6	27	23,9	29	27,4		99	28,2
High	50	37,9	41	36,3	28	26,4		119	33,9
ADL (mean, SD)	6,0	0,3	6	0	6,0	0,2	*0,226*	6,0	0,2
IADL (mean, SD)	7,7	0,7	7,8	0,6	7,8	0,6	*0,502*	7,8	0,7
Global cognitive function (mean, SD)^#^	27,4	1,9	27,1	2,2	27,3	2,1	*0,504*	27,3	2,0
MCI°	23	17,4	27	24,1	30	28,6	*0,120*	80	22,9
Depressive symptoms^	16	12,1	25	22,1	24	22,6	*0,056*	65	18,5

Mild Cognitive Impairment, ^#^Mini-mental-state-examination, ^CES-D ≥ 16. Italic values indicating comparison of the participants’ characteristics among tertiles of the fear score.

**TABLE 2:** Follow-up characteristics of participants by tertiles of fear score (*N* = 351).

Variables collected during phone interview	Low (score ≤ 4) *N* = 102, 22.6%		Medium (score 5-7) *N* = 106, 30.2%		High (score 8+) *N* = 103, 22.8%		*P–value*	All *N* = 351	
Altered consciousness	2	1,5	5	4,4	4	3,8	*0,386*	11	3,1
Fever	3	2,3	8	7,1	5	4,7	*0,198*	16	4,6
Cough	10	7,6	13	11,5	14	13,2	*0,343*	37	10,5
Sore throat	11	8,3	10	8,9	17	16,0	*0,117*	38	10,8
Rhinorrhoea	10	7,6	11	9,7	22	20,8	*0,005*	43	12,3
Headache	2	1,5	11	9,7	11	10,4	*0,009*	24	6,8
Myalgia	11	8,3	9	8,0	17	16,0	*0,087*	37	10,5
Olfactory and taste disorders	0	0	1	0,9	3	2,8	*0,118*	4	1,1
Shortness of breath	7	5,3	9	8,0	6	5,7	*0,661*	22	6,3
Chest pain	4	3,0	6	5,3	6	5,7	*0,562*	16	4,6
Tachycardia	6	4,6	2	1,8	10	9,5	*0,032*	18	5,1
Gastrointestinal disturbances	4	3,0	9	8,0	7	6,7	*0,223*	20	5,7
Conjunctivitis	12	9,1	13	11,5	10	9,5	*0,806*	35	10,0
Pneumonia	1	0,8	3	2,7	1	1,0	*0,407*	5	1,4
Anti-inflammatory drugs	37	28,0	44	39,6	44	41,9	*0,053*	125	35,9
Anxiety medication and/or sedatives	23	17,4	18	15,9	19	17,9	*0,919*	60	17,1
Supplements (e. g,, vitamins)	43	32,6	50	44,3	41	38,7	*0,171*	134	38,2
Antibiotics	19	14,4	14	13,4	20	18,9	*0,392*	53	15,1
Antihistamines	10	7,6	9	8,0	8	7,6	*0,991*	27	7,7
Anti-pneumococcal vaccine	9	6,8	10	8,9	14	13,2	*0,237*	33	9,4
Flu vaccine	77	58,3	73	64,6	70	66,0	*0,416*	220	62,7
Hospitalised for COVID19	2	1,5	2	1,8	1	1,0	*0,871*	5	1,5
NPS test							*0,580*		
No, never	131	99,2	112	99,1	105	99,1		348	99,2
Yes, always negative	0	0	1	0,9	1	0,9		2	0,6
Yes, always positive	1	0,8	0	0	0	0		1	0,3
Weekly outing, never	91	68,9	74	65,5	61	57,6	*0,181*	226	64,4
Use of public transport, never	12	5,3	7	6,2	1	0,9	*0,120*	15	4,3
Emergency number/doctor contacts	7	5,3	10	8,9	8	7,6	*0,549*	25	7,1
Contact COVID19 case	9	6,8	6	5,3	7	6,6	*0,876*	22	6,3

Italic values indicating comparison of the participants’ characteristics among tertiles of the fear score.

**TABLE 3:** Multinomial logistic regression model between baseline characteristics of participants and the tertiles of score fear.

	Medium (5-7) vs. Low (≤ 4)		High (8+) vs. Low (≤ 4)	
		
	aOR*	95% CI	aOR*	95% CI
Women	1.56	0.89–2.74	1.91	1.04–3.51
Age, years (mean, SD)	1.00	0.93–1.02	0.93	0.88–0.98
Education				
High school or more	1 (ref.)		1 (ref.)	
Middle school	1.04	0.56–1.90	1.12	0.59–2.15
Illiterate or primary school	0.92	0.41–2.08	1.45	0.64–3.29
Smoking status				
Never	1 (ref.)		1 (ref.)	
Former or current	0.89	0.51–1.55	0.58	0.32–1.04
Body mass index (mean, SD)	1.00	0.94–1.06	1.06	1.00–1.14
Polypharmacy (>5 drugs/day)	1.39	0.71–2.73	1.52	0.75–3.05
Leisure activities				
Low	1 (ref.)			
Moderate	0.58	0.29–1.15	0.63	0.31–1.26
High	0.74	0.39–1.42	0.51	0.25–1.03
Anti-pneumococcal vaccine	1.26	0.47–3.38	1.88	0.72–4.93
Depressive symptoms^	1.74	0.85–3.58	1.61	0.76–3.41
MCI*	1.47	0.72–3.02	1.90	0.92–3.96

Results of baseline characteristics are reported as adjusted Odds Ratio (aOR) with 95% confidence interval (CI). Mild Cognitive Impairment; ^CES-D ≥ 16.

**TABLE 4:** Binary logistic regression model between baseline characteristics of participants and fear of contagion (medium-high fear vs. no or low fear) compared with peers (model A), for themselves (model B, and for family members (model C).

	Model A fear of contagion compared with peers (*N* = 67, 19.1%)		Model B fear of contagion for themselves (*N* = 116, 33.1%)		Model C fear of contagion for family members (*N* = 199, 56.7%)	
			
	aOR	95% CI	aOR*	95% CI	aOR*	95% CI
Women	1.15	0.62–2.14	1.29	0.75–2.23	1.40	0.86–2.28
Age, years	1.01	0.97–1.07	0.94	0.89–0.98	0.94	0.90–0.98
Education						
High school or more	1 (ref.)		1 (ref.)		1 (ref.)	
Middle school	1.04	0.54–1.98	1.38	0.78–2.45	1.36	0.79–2.31
Illiterate or primary school	0.97	0.42–2.22	1.88	0.92–3.81	1.59	0.79–3.19
Smoking status						
Never	1 (ref.)		1 (ref.)		1 (ref.)	
Former or current	0.83	0.45–1.51	0.86	0.75–2.46	0.64	0.39–1.03
Body mass index	1.00	0.95–1.07	1.08	1.02–1.14	1.04	0.98–1.09
Polypharmacy (>5 drugs/day)	1.28	0.65–2.50	1.36	0.75–2.45	1.40	0.78–2.51
Leisure activities						
Low	1 (ref.)		1 (ref.)			
Moderate	0.57	0.27–1.18	0.57	0.31–1.06	0.89	0.49–1.60
High	0.86	0.44–1.69	0.46	0.23–0.85	0.93	0.53–1.65
Anti-pneumococcal vaccine	2.60	1.14–5.90	1.22	0.53–2.80	3.09	1.23–7.74
Depressive symptoms^	1.10	0.54–2.25	1.15	0.61–2.17	2.38	1.25–4.52
MCI*	0.68	0.32–1.40	2.55	1.39–4.70	1.44	0.78–2.68

Results of baseline characteristics are reported as adjusted Odds Ratio (aOR) with 95% confidence interval (CI). Mild Cognitive Impairment; ^CES-D ≥ 16.

## Discussion

The present paper investigated the association between pre-existing mental health disorders and fear of COVID-19 among community-dwelling older adults. The main results indicate that individuals with pre-pandemic neurocognitive disorders and depressive symptoms had a high perception of fear showing different reactions to domains of fear; furthermore, impaired executive function domain increased level of fear. We also found that some socio-demographic, clinical and behavioral conditions were associated with fear of COVID-19.

The findings of the study indicate that the COVID-19 pandemic created moderate fear among the older population with a mean fear score of 5.7 (fear score ranging between 0 and 12). Only a few studies were conducted focusing on the older adult cohorts and because of the different fear assessment tools used, comparisons are limited. In a sample of older Bangladeshi adults aged ≥60 years, the authors reported that the COVID-19 pandemic created high fear, with a mean fear score of 19.4 (range between 7 and 35) (29). Similarly, fear perception was found quite high (mean fear score of 19.3, range between 7 and 35), among 500 people aged 60 years and more in Poland (30). Doshi et al. (31) observed that in an Indian population aged more than 60 years, a significantly higher number of the participants reported low fear (54.8%). We also found that fear prevailed when referring to family members over fear for oneself in our older sample, as we previously observed in the web-based EPICOVID19 survey although it was conducted in a younger sample (28).

In our study, the occurrence of MCI was found to be 22.9%, a percentage that quite overlapping to another Italian population-based study performed on 2337 individuals over 65 years residents in Northern Italy (prevalence 21.6%) (32). Depressive symptomatology occurred in 18.5% of the study sample. The official data from the report of Istat – National Institute of Statistics indicate a prevalence of depression of 14.9% in Italians aged 64 or more. The Italian Faenza project, which included 359 subjects aged 74 years and older, reported an overall prevalence of 25.1% (^2^2018, July, 26). However, these comparisons should be taken with caution, because of the inherently different assessment methods used and sampling strategy.

Our findings showed that pre-pandemic mental health disorders were positively associated with a heightened fear of COVID-19. To the best of our knowledge, the present study represents the first attempt to provide data on the association between pre-existing neurocognitive decline and depression and different domains of fear of the COVID-19 outbreak among older people also exploring the role of specific cognitive tasks. However, some similarities with other research can be found. Three studies conducted during the lockdown in 2020 among patients (11, 12) and community-dwelling (13) older seniors with pre-existing neurocognitive disorders showed worsening neuropsychiatric traits, such as anxiety, agitation and apathy that reduce flexibility and coping abilities. Adsmundson et al. (33) analyzed data collected from Canada and the United States using an online survey between March 21 and April 1, 2020 and reported that people with pre-existing mental health disorders, like depressive and anxiety symptoms, have higher stress levels than the general population, and more irritability and anxiety and less coping strategies during pandemic restrictions. These consequences created a sense of impotence that increases different kinds of negative emotions. In a cross-sectional study of older community-dwelling older adults, affective symptoms (which include both depressive and anxiety symptoms), were associated with a heightened fear of COVID-19 (5).

Our findings should be interpreted in the light of the known interplay between cognition and emotions (34, 35). Older people with cognitive decline are more susceptible to changes and this is a factor that can feel different kinds of emotions like agitation, frustration and fear. Emotions, including fear, have fundamental constituents that are cognitive in nature. In particular, some higher cognitive functions (e.g., working memory/updating, attention/inhibitory control, and cognitive flexibility) – named with the umbrella term “executive functions” – are involved in and contribute to the cognitive regulation of emotions (36). Therefore, alterations in these cognitive functions may affect people’s ability to regulate emotions and mood states. In detail, the emotional dysregulation experienced by people with mood disorders, such as depression, can profoundly affect also those key cognitive processes involved in the cognitive regulation of emotions (37, 38), with consequent maladaptive response to negative events as fear due to pandemic. Likewise, the cognitive deficits that characterize people with MCI or confirmed dementia (39) may influence processes involved in the development of adequate coping strategies, personal beliefs and emotional responses (40, 41). In particular, the prefrontal cortex (PFC) plays a crucial role as the chief executive officer of the brain, controlling the highest level of cognitive and emotional processes (36). For these reasons, people with neurocognitive disorders and depressive symptoms would tend to show greater fear of COVID-19 infection, as our results also suggest. When we explored cognitive abilities associated with coping with the fear of the pandemic, interestingly we found that individuals with executive dysfunction had a high level of fear and, particularly, fear of being infected by the virus. It would appear that the presence of cognitive impairment has an impact on the choice of coping response and particularly, deficits in executive functions have been associated with less use of problem-solving and greater use of coping strategies associated with poorer outcomes (40). In fact, coping strategies associated with a better outcome, such as problem-focused, often require relatively high-level cognitive functioning, including cognitive flexibility, adaptation, and planning skills, particularly in contrast to some emotion-focused strategies such as passive avoidance (41).

We also observed other factors associated with the fear of COVID-19. Women had higher levels of fear compared with men. This is completely in line with previous studies indicating that women are more worried about the outbreak of COVID-19 and present more anxiety and stress (28, 42). Age increase was inversely associated with the fear of COVID-19. This observation might be counterintuitive because, since the beginning of the pandemic, older people have been recognized as the frailer and more vulnerable to COVID-19 infection and death (5). Therefore, they should be more worried about their health status. Nevertheless, our findings are supported by previous studies demonstrating that very old people show a capacity to adapt to adversities and difficulties with strategies by focusing more on the positive aspects of the situations (43). This means that older individuals might have a higher sense of resilience in coping with the pandemic as compared with the younger generations, possibly due to the previous experiences they have had to deal with in their life (e.g., other epidemics, war, and post-war) (44).

In addition to that, our data suggest that older persons who were engaged in leisure activities (social, mental and physical) throughout the life course and prior to the pandemic, had low fear perception. Once again, these findings emphasized that older people are more resilient and able to cope with stress and fear as compared with young people. Indeed, the evidence indicates that participation in recreational and leisure activities improves mental health by reducing anxiety, depression and cognitive impairment (45, 46). Speculatively, those persons who were active during their life could have maintained their virtuous behaviors even during the lockdown and this may have provided mental health benefits for older adults during the COVID-19 pandemic (47).

In line with previous evidence, we found that older people overweight/obese individuals (48) showed a high level of anxiety and fear. Since the beginning of the pandemic, obesity, which is a condition associated with underlying risk factors for COVID-19, including hypertension, dyslipidaemia, type 2 diabetes and chronic kidney or liver disease (49), have been consistently reported to be factors that increased the risk of mortality for COVID-19. This health negative consequence makes this sub-group of persons more susceptible to suffering from fears about their health and associated psychological distress.

Our findings also suggest that the anti-pneumococcal vaccine was positively associated with fear of being infected compared with peers and fear of contagion for family members. These findings are in line with those we published during the same period of observation in the EPICOVID19 study (28). A possible explanation might reside in the fact that, at that time when no COVID-19 treatment was available, vaccination for other respiratory diseases was considered the unique crucial preventive measure to face the infection (50).

Our data also seem to suggest that former or current smokers had a low fear perception as compared with non-smokers. In a previous article, Herbec and colleagues (51) reported that current smokers showed optimism bias when considering their own behavior of smoking, as a factor that increased the risk for severe COVID-19 symptoms. This is in line with previous studies demonstrating that smokers have a misperception about the harms of smoking effects and tend to underestimate the extent to which smoking elevates their risks of developing diseases (52). Based on these considerations, we might speculate that smokers who continue to smoke even in old age, tend to have a low-risk perception regarding their health status and possibly, a low fear perception regarding the pandemic.

### Limits and strengths

This study presents some limitations that we have to consider. Firstly, the set of questions used to assess fear in the sample was not validated and standardized making a comparison across studies difficult. However, the questions were extracted from the EPICOVID19 questionnaire already used in previous papers (27, 28). Secondly, although we controlled for several potential confounders, we cannot completely rule out the possibility of residual confounding due to unmeasured factors. The present study also has several strengths: the study provides data from a community-based older population; an exhaustive assessment of the participant’s cognitive function, and clinical and behavioral factors using validated questionnaires and scales administered by trained personnel that reduces recall bias; these information were collected before the beginning of the pandemic, thus limiting reverse causation; the combined use of the standardized neuropsychological battery and the clinical examination to confirm MCI cases that enhance the sensitivity of the diagnosis; the high response rate to the phone survey (91%) that reduce the selection bias.

## Conclusion

This study identified the characteristics of individuals who are more likely to react fearfully toward the COVID-19 pandemic in Italy. Older adults experience different levels and types of fear reactions, particularly when suffering from neurocognitive deficits and depressive symptoms. These results, in agreement with other authors, highlight the need to pay attention to the psychological effects of the outbreak of COVID-19 especially among vulnerable subgroups of people. Specific strategies and interventions should be targeted to support the mental wellbeing of these individuals in addition to the existing resources within primary healthcare settings.

## NutBrain study group

Fulvio Adorni, Sara Bernini, Silvia Conti, Maria Lea Correa Leite, Alfredo Costa, Matteo Cotta Ramusino, Nithiya Jesuthasan, Massimo Musicco, Orietta Pansarasa, Federica Prinelli, Elena Perdixi, Anna Pichiecchio, Marco Severgnini, Elena Sinforiani.

## Data availability statement

Datasets are available on request: The raw data supporting the conclusions of this article will be made available by the authors, without undue reservation.

## Ethics statement

The studies involving human participants were reviewed and approved by Medical Ethical Committee of Pavia, Italy. The patients/participants provided their written informed consent to participate in the NutBrain study. Due to the pandemic, informed consent to the phone interview and the use of the data was provided orally.

## Author contributions

FP conceived and designed the study, acquired funding and ethics approval, had full access to all data, had responsibility for the data collection integrity, and performed the statistical analysis. EP and FP drafted the original version of the manuscript. SB, NJ, and SC made substantial contributions to the conception and design of the study, collected data, helped to draft the manuscripts, and interpreted the results. AC and MC made substantial contributions to the conception and design of the study, interpreted the results, and reviewed the manuscript. All authors reviewed the manuscript, interpreted the results, and agreed to be accountable for all aspects of the work in ensuring that questions related to the accuracy or integrity of any part of the work are appropriately investigated and resolved.

## Footnotes

1
http://www.nutbrain.it/study-project/

2
https://www.istat.it/en/archivio/219812

## Abbreviations

COVID-19: Coronavirus disease
NutBrain: Nutrition, gut microbiota, and brain aging
MCI: Mild Cognitive Impairment
CRIq: Cognitive Reserve Index questionnaire
ADL: Katz Index of Independence in Activities of Daily Living
IADL: Instrumental Activities of Daily Living scale
CES-D: Center for Epidemiologic Studies Depression scale
SD: Standard deviation
ORs: Odds ratios
CIs: Confidence Intervals
FAB: Frontal Assessment Battery
FCSRT: Free and Cued Selective Reminding Test
ROCF: Rey-Osterrieth Complex Figure Test.
