# Supplementary material for: Pre-existing mental health disorders and fear of COVID-19 pandemic: Data from a phone survey in community-dwelling older adults recruited in the NutBrain study
Source: Front Psychiatry. 2022 Nov 7;13:995308. doi: 10.3389/fpsyt.2022.995308 (PMC9676658; doi:10.3389/fpsyt.2022.995308)
Supplement: Supplementary file 1 [file Data_Sheet_1.docx]

**Supplementary material**

The standardized neuropsychological examination included:

1. *global cognitive functioning*: Mini-Mental State Examination (MMSE) (1): score ranges from 0 to 30 points, with a lower score indicative of worse performance;
2. *memory functions*: Free and Cued Selective Reminding Test (FCSRT) (2), Logical Memory Test (3), Rey-Osterrieth Complex Figure Test (ROCF) – delay recall (4);
3. *attention*: Trail Making Test part A (TMT A) (8)
4. *executive functions*: Frontal Assessment Battery (FAB) (5), phonemic (6) and semantic verbal fluency (7), Trail Making Test part B (TMT B) (8);
5. *language*: Picture Naming Test (9);
6. *visuospatial abilities*: Rey-Osterrieth Complex Figure Test (ROCF) – copy (4).

**Supplementary Table S1.** **Multinomial logistic regression model between impaired neuropsychological test and the tertiles of score fear.**

| **Impaired vs normal values** | **Medium**  **(5-7) vs Low (≤ 4)** | | **High**  **(8+) vs Low (≤ 4)** | |
| --- | --- | --- | --- | --- |
|  | **aOR*** | **95%CI** | **aOR*** | **95%CI** |
| FAB | 1.45 | 0.58-3.61 | 3.28 | 1.37-7.84 |

Results are reported as adjusted Odds Ratio (aOR) with 95% confidence interval (CI). *Model adjusted for age, gender, education, polypharmacy, smoking status, leisure activities, body mass index, and anti-pneumococcal vaccination.

**Supplementary Table S2.** **Binary logistic regression model between impaired neuropsychological test and fear of contagion for themselves and family members**

| **Impaired vs normal values** | **Fear of contagion for themselves** | |
| --- | --- | --- |
| **Model** | **aOR*** | **95%CI** |
| FCSRT immediate cued recall | 2.07 | 0.17-5.81 |
| ROCF delay recall | 2.85 | 0.83-9.83 |
| FAB | 3.39 | 1.61-7.17 |
| ROCF copy | 0.73 | 0.10-1.70 |

Results are reported as adjusted Odds Ratio (aOR) with 95% confidence interval (CI). *Model adjusted for age, gender, education, polypharmacy, smoking status, leisure activities, body mass index, anti-pneumococcal vaccination and including all neuropsychological tests simultaneously.

**References**

1. Magni E, Binetti G, Bianchetti A, et al.: Mini-Mental State Examination: a normative study in Italian elderly population Eur J Neurol 1996 and 3:198–202.

2. Frasson P, Ghiretti R, Catricalà E, et al.: Free and cued selective reminding test: an Italian normative study Neurol Sci 2011 and 32:1057–1062.

3.Novelli G, Papagno C, Capitani E, Laiacona M, Cappa SF VG: Tre test clinici di memoria verbale a lungo termine. Taratura su soggetti normali. Arch di Psicol Neurol Psychiatry 1986 and 47:278–296.

4.Caffarra P, Vezzadini G, Dieci F, et al.: Rey-Osterrieth complex figure: normative values in an Italian population sample Neurol Sci 2002 and 22:443–447.

5. Appollonio I, Leone M, Isella V, et al.: The Frontal Assessment Battery (FAB): normative values in an Italian population sample Neurol Sci 2005 and 26:108–116.

6. Carlesimo GA, Caltagirone C, Gainotti G, et al.: The Mental Deterioration Battery: Normative Data, Diagnositc Reliability and Qualitative Analyses of Cognitive Impaiment Eur J Neurol 1996 and 36:378–384.

7. Novelli G, Papagno C, Capitani E, Laiacona M, Cappa SF VG: Tre test clinici di memoria verbale a lungo termine. Taratura su soggetti normali. Arch di Psicol Neurol Psychiatry 1986 and 47:278–296.

8. Giovagnoli AR, Del Pesce M, Mascheroni S, et al.: Trail making test: normative values from 287 normal adult controls Ital J Neurol Sci 1996 and 4:305–309.

9. Sartori G, Job R: The oyster with four legs: A neuropsychological study on the interaction of visual and semantic information Cogn Neuropsychol 1988 and 5:105–132.
